# Functional Nucleic Acid Probes Based on Two-Photon for Biosensing

**DOI:** 10.3390/bios13090836

**Published:** 2023-08-23

**Authors:** Kefeng Wu, Changbei Ma, Yisen Wang

**Affiliations:** 1 GBA Branch of Aerospace Information Research Institute, Chinese Academy of Sciences, Guangzhou 510700, China; 2 Guangdong Provincial Key Laboratory of Terahertz Quantum Electromagnetics, Guangzhou 510700, China; 3 School of Life Sciences, Central South University, Changsha 410013, China

**Keywords:** DNA nanotechnology, two-photon, bioimaging, fluorescent probe, biomolecule, light-activated, RNA

## Abstract

Functional nucleic acid (FNA) probes have been widely used in environmental monitoring, food analysis, clinical diagnosis, and biological imaging because of their easy synthesis, functional modification, flexible design, and stable properties. However, most FNA probes are designed based on one-photon (OP) in the ultraviolet or visible regions, and the effectiveness of these OP-based FNA probes may be hindered by certain factors, such as their potential for photodamage and limited light tissue penetration. Two-photon (TP) is characterized by the nonlinear absorption of two relatively low-energy photons of near-infrared (NIR) light with the resulting emission of high-energy ultraviolet or visible light. TP-based FNA probes have excellent properties, including lower tissue self-absorption and autofluorescence, reduced photodamage and photobleaching, and higher spatial resolution, making them more advantageous than the conventional OP-based FNA probes in biomedical sensing. In this review, we summarize the recent advances of TP-excited and -activated FNA probes and detail their applications in biomolecular detection. In addition, we also share our views on the highlights and limitations of TP-based FNA probes. The ultimate goal is to provide design approaches for the development of high-performance TP-based FNA probes, thereby promoting their biological applications.

## 1. Introduction

Nucleic acids have gained prominence as a fundamental framework of functionally diverse and versatile molecular probes due to their unique molecular recognition capabilities, programmable base sequences, convenient synthesis and modification, and stimuli-triggered responsiveness [1,2,3,4]. Functional nucleic acids (FNA) mainly include aptamers (which selectively bind with numerous biological target molecules) [5,6,7,8], DNAzymes (which catalyze the cleavage of various substrates like enzymes) [9,10,11], and chemically modified nucleic acids (which possess all kinds of specific functions and can response to stimuli factors) [12,13,14]. They can be obtained by the systematic evolution of ligands by exponential enrichment (SELEX) or chemical reactions and exhibit much specific recognition capability for a variety of unique biological applications [15,16,17,18,19].

Based on the many advantages of FNA molecules, extensive efforts have been made in the development of FNA probes used in detecting a diverse array of analytes [20], such as RNAs [21,22,23,24,25], proteins [26,27,28], small molecules [29,30,31], and metal ions [32,33]. These functional nucleic acid probes mainly use the specific recognition between the base sequence and the target molecule and combine the techniques of in situ hybridization, fluorescence resonance energy transfer (FRET), and signal amplification strategy to investigate dynamic changes and interaction processes at the molecular level; they have shown significant superiority in unraveling the fundamental mechanisms of pathophysiological processes and facilitating disease diagnosis [34,35,36,37]. As a result, the ongoing advancement and utilization of FNA probes hold great promise for advancing our understanding of biology and improving diagnostic approaches [38,39,40].

Typically, most FNA probes were primarily designed based on one-photon (OP) that is required to be excited/activated by a short wavelength light source in the ultraviolet or visible (UV/Vis) regions [41,42]. OP-based FNA probes possess advantages such as high sensitivity and selectivity, programmability, ease of synthesis and modification, and real-time monitoring, which have led to their widespread application in biomedical research, nanotechnology, and materials science [43,44,45,46,47]. However, the inevitable disadvantages of OP light sources, including shallow tissue penetration, photobleaching, phototoxicity, and autofluorescence from biological systems, greatly restrict the development of OP-based FNA probes in various organelles and deep tissues.

In comparison with OP-based FNA probes that employ a short wavelength light source, FNA probes based on two-photon (TP) can be excited/activated by a long wavelength femtosecond laser [48,49,50]. The theory of TP absorption was initially introduced by Maria Goeppert-Mayer in the 1930s [51] and, with the advent of femtosecond laser technology, researchers began investigating this instrumentation in the 2000s. TP for biosensing use near-infrared (NIR) photons as the light source, which can achieve accurate regulation of the detection process of FNA probes. NIR light causes less damage to cells, reduces autofluorescence from biological tissue, allows for deeper tissue penetration [52,53,54,55], and gives out negligible autofluorescence in biological matrices [56,57]. The above advantages make TP-based FNA probes more promising for various biological applications when compared to conventional OP-based FNA probes [58,59].

**Figure 1:**
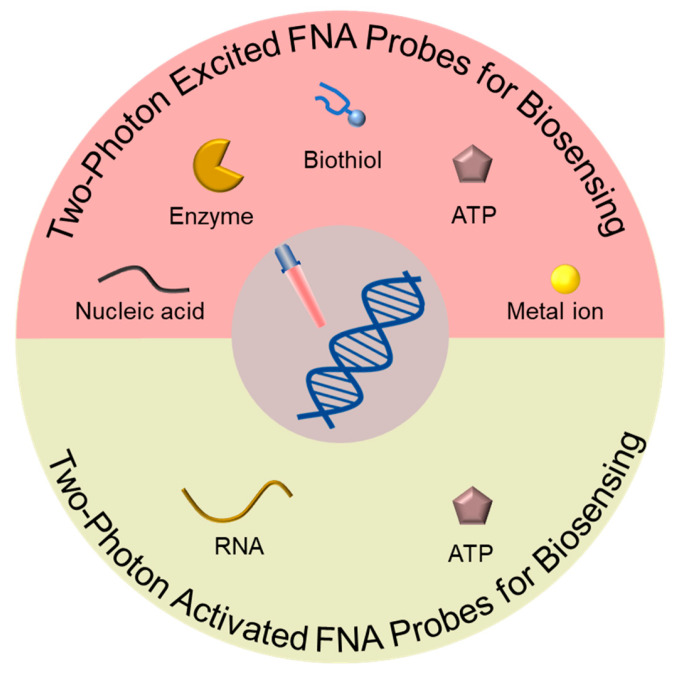
Schematic overview of TP-based FNA probes used for biosensing.

This review (Figure 1) provides a comprehensive overview of the designs and applications of TP-based FNA probes used as biosensors. Initially, we emphasize the utilization of TP-excited FNA probes in the detection of various analytes, including nucleic acids, enzymes, biothiols, ATP, and metal ions. Subsequently, we delve into TP-activated FNA probes and their specific applications in RNA and ATP detection. Finally, we discuss the existing challenges and future outlooks regarding TP-based FNA probes used in the field of biomedical research.

## 2. Two-Photon Excited FNA Probes for Biosensing

FNA probes based on one-photon excitation (OPE) suffer from limitations such as limited tissue penetration depth, photodamage, and photobleaching. To overcome these challenges, two-photon excitation (TPE) fluorescence imaging has become an optimal approach for enhancing sensing capabilities in living cells and tissues. In contrast to the prevailing construction strategy of OPE-based FNA probes, TPE-based FNA probes constructed by introducing TP dye as the signal reporter can effectively eliminate the problems related to shallow imaging depth and background fluorescence interference in biological matrices while using an NIR excitation wavelength that provides increased penetration depth and reduced photodamage. Therefore, the applications of TPE-based FNA probes are extensive in the field of biosensing, allowing for the detection of biomolecules such as nucleic acids, enzymes, biothiols, ATP, and metal ions. By investigating the dynamics and interactions between molecules, the underlying mechanisms of pathophysiological processes can be better attained, thereby advancing disease diagnosis and therapeutic monitoring.

### 2.1. Two-Photon Excited FNA Probes for Nucleic Acid Analysis

MicroRNAs (miRNAs) are short noncoding nucleic acid fragments, typically comprising 21–23 nucleotides, that play crucial roles in gene expression regulation and cellular functions [60]. MiRNAs can bind to target mRNA, leading to its inhibition or degradation, thereby regulating the expression levels of genes [61]. This regulatory mechanism has important functions in various biological processes, including maintaining cellular homeostasis, developmental regulation, cell proliferation, and apoptosis [62,63,64]. Due to the specific expression patterns of miRNAs in different tissues and disease states, assaying miRNA levels in body fluids can contribute to early disease diagnosis, prognostic assessment, and monitoring treatment response [65,66]. Consequently, monitoring the dynamic expression of miRNAs in living cells holds significant value in gaining insights into miRNA-associated cellular processes and facilitating disease diagnosis.

**Figure 2:**
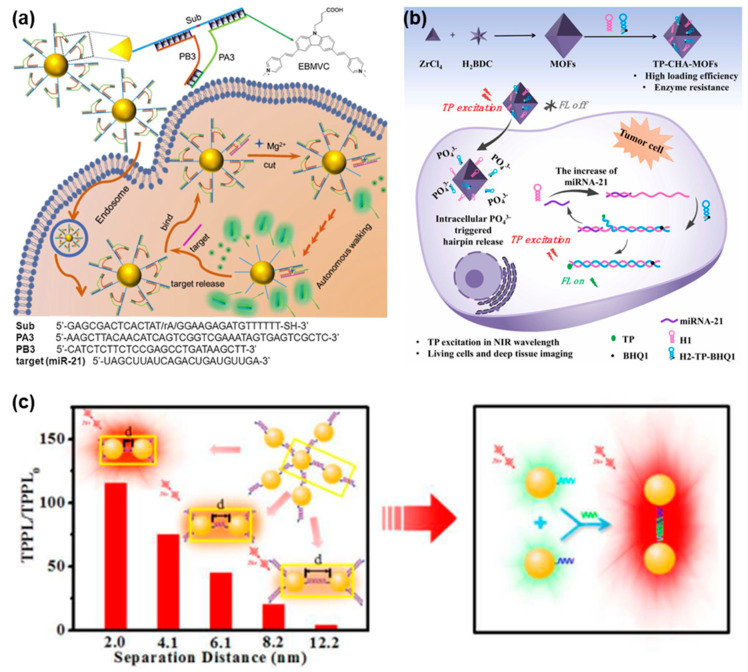
FNA probes based on TPE for nucleic acid analysis. (**a**) Schematic illustration of a TPE sensing platform based on spherical DNAzyme for intracellular miRNA assay. Adapted with permission from [67]. (**b**) Schematic illustration of the synthesized TP-CHA-MOFs for miRNA imaging in tumor cells. Adapted with permission from [68]. (**c**) Schematic illustration of DNA-induced coupling of AuNPs and TPPL enhancement for DNA sequence detection. Adapted with permission from [69].

In general, TPE-based FNA probes were designed by incorporating TP dye molecules that could be excited by a TP laser to produce fluorescence signals. As shown in Figure 2a, Wang et al. constructed a spherical nucleic acid (SNA)-based fluorescent probe by incorporating TPE and DNAzyme for the highly selective and sensitive analysis of target miRNAs in living cells [67]. Their approach involved functionalizing gold nanoparticles (AuNPs) with substrate strands capable of hybridizing into two split DNAzyme fragments. In the formation of the TPE-SNA nanoprobe, the substrate/split-DNAzyme duplexes were incorporated with multiple TP dye molecules, leading to a state where fluorescence emission was turned off. When target miRNA was present, the conformational rearrangement of the split DNAzyme would be induced and their catalytic ability could be activated, thus leading to the cleavage of the substrate strands and the subsequent release of the TP dye molecules. Simultaneously, the liberated target miRNA could spontaneously form a hybrid with another split DNAzyme fragment, initiating additional cleavage reactions. This sequential hybridization-activated cleavage process gradually dissociated the TP dye molecules from the surface of the AuNPs. As a result, a cascade of TPE fluorescence emission occurred, amplifying the fluorescence signals for miRNA detection. In addition, Yang et al. developed a TPE-based nanoprobe for imaging intracellular miRNA by employing metal-organic frameworks (MOFs) as the nanocarrier along with hairpins to amplify TP fluorescence signals (Figure 2b) [68]. Hairpin 2 (H2) was engineered with TP fluorophore and BHQ1 quencher modifications, where the proximity of BHQ1 in the hairpin structure led to the fluorescence quenching of TP fluorophore. After being delivered into cells by MOFs, hairpin 1 (H1) and H2 could be triggered by intracellular miRNA, leading to the formation of an H1–H2 duplex through a catalytic hairpin assembly (CHA) reaction. This contributed to the separation of the TP fluorophore from the BHQ1 quencher, resulting in the activation of TP fluorescence and the release of the miRNA. The released miRNA could further trigger the formation of additional H1–H2 duplexes, enabling the amplified target miRNA detection. The incorporation of TP fluorophore facilitated the effective imaging of miRNA in both living tumor cells and deep tumor tissues, achieving a notable penetration depth of 160 µm. This TPE-based nanoprobe demonstrated promising capabilities for sensitive and specific miRNA detection in complex biological environments.

To accurately measure the length of DNA molecules, Yuan et al. conducted a study where they prepared assemblies of DNA-tuned AuNPs with controlled separation distances to investigate the impact of plasmon coupling strength and particle size on two-photon photoluminescence (TPPL) enhancement (Figure 2c) [69]. They observed that decreasing the separation distance between the DNA-coupled nano-assemblies resulted in a significant increase in TPPL intensity. Based on this finding, they developed a TP sensing scheme for detecting DNA sequences by utilizing the DNA-induced coupling of AuNPs and TPPL enhancement. The developed method exhibited exceptional sensitivity, achieving a limit of detection (LOD) as low as 2.9 pM, enabling the detection of DNA sequences at very low concentrations. Additionally, it demonstrated excellent selectivity against single-stranded DNA (ssDNA) with mismatched bases, allowing for the easy differentiation of even a single mismatch at room temperature. Moreover, the researchers highlighted the potential extension of this method to DNA detection in living cells or in vivo, leveraging the unique advantages of TPE. Therefore, the TPPL-based method shows promise for sensitive and selective DNA detection in biological systems.

### 2.2. Two-Photon Excited FNA Probes for Enzyme Analysis

Enzymes are specific and efficient biocatalysts, most of which are proteins produced by living cells that perform essential roles in living systems by accelerating biochemical reactions through their highly efficient catalytic activities [70,71,72]. Due to their specificity and efficiency, enzymes are essential for maintaining the proper functioning of biological processes. However, abnormal enzyme activity could have detrimental effects and contribute to various diseases [73,74,75,76]. By assaying the activity of specific enzymes or their levels in tissues, cells, or bodily fluids, we can assess the status and functionality of specific biological processes [77]. For example, measuring the activity of liver enzymes provides information about liver function, liver diseases, and drug metabolism. Enzyme detection can also be used to monitor changes in processes such as cell proliferation, apoptosis, signal transduction pathways, and gene expression [78,79]. Hence, there is a need to create and advance probes capable of directly detecting enzyme activity in vivo, particularly with a focus on non-invasive and real-time methods.

**Figure 3:**
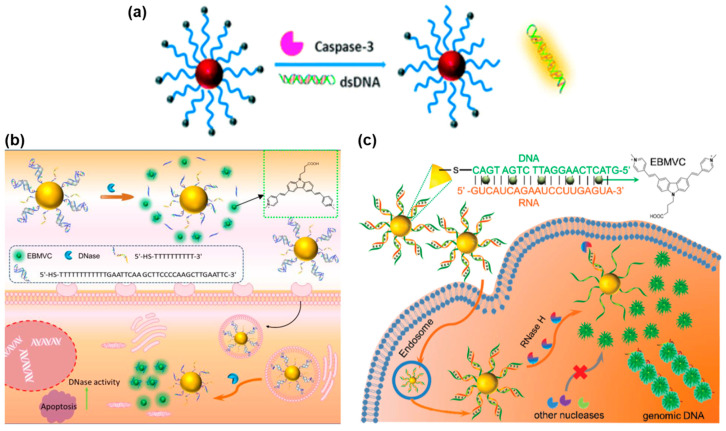
FNA probes based on TPE for enzyme analysis. (**a**) Schematic illustration of the AuNPs/peptide-TP dye nanoprobe for assaying caspase-3. Adapted with permission from [80]. (**b**) Schematic illustration of the TPE-based nanoprobe for imaging DNase. Adapted with permission from [81]. (**c**) Schematic illustration of the SNA-based TPE sensor for detecting RNase H. Adapted with permission from [82].

According to the different hydrolysis characteristics of enzymes, different nucleic acid sequences that responded to the hydrolysis of enzymes were designed on the FNA probes. As shown in Figure 3a, Ge et al. developed a FRET nanoprobe that was excited by a TP laser and successfully applied in imaging caspase-3 by assembling TP dye-labeled peptides on the surface of AuNPs [80]. This study is the first successful application of an AuNP/peptide-TP biosensor for evaluating caspase-3 activity in both live cells and liver tissues that have undergone ischemic reperfusion surgery. In a separate study, Wang et al. reported a TPE-based nanoprobe used for assessing DNase activity both in vitro and ex vivo (Figure 3b) [81]. This nanoprobe primarily consisted of a AuNP core acting as the cellular transporter and fluorescence quenching motif, along with TP dyes incorporated into the double-stranded DNA (dsDNA) to act as the reporter motif. When DNases were present, the dsDNA immobilized on the surface of AuNPs underwent hydrolysis, releasing a large amount of TP dyes. Consequently, the TP dyes exhibited fluorescence recovery, allowing for the measurement of DNase activity. This TPE-based nanoprobe has been successfully applied in the monitoring of DNase activity in living cells and in vivo. Furthermore, Wang et al. also reported a TPE-based nanoprobe for detecting RNase H activity in living cells and ex vivo tissues, as depicted in Figure 3c [82]. This nanoprobe involved binding TP dyes to spherical nucleic acids (SNA) with a DNA/RNA duplex corona and a AuNP core. The developed probe offers a convenient means for detecting RNase H activity, which is expected to pave the way for further research in the field of RNase H.

### 2.3. Two-Photon Excited FNA Probes for Biothiol Analysis

Biothiols, which encompass thiol-containing amino acids and peptides, serve as vital structural or functional constituents in many proteins and peptides and fulfill various essential functions in biological systems [83,84]. GSH, known as the predominant intracellular non-protein thiol in the cytoplasm, acts as a crucial reducing agent involved in various cellular functions, such as maintaining intracellular redox activity, facilitating xenobiotic metabolism, enabling intracellular signal transduction, and regulating gene expression [85,86,87]. Measuring the levels of biothiols provides valuable information to assess oxidative-reductive status, antioxidant capacity, and the risks and progression of certain diseases, such as cardiovascular diseases, cancer, and neurodegenerative diseases [88]. Given their significance in physiological activities, monitoring biothiol molecules has garnered considerable interest [89,90].

**Figure 4:**
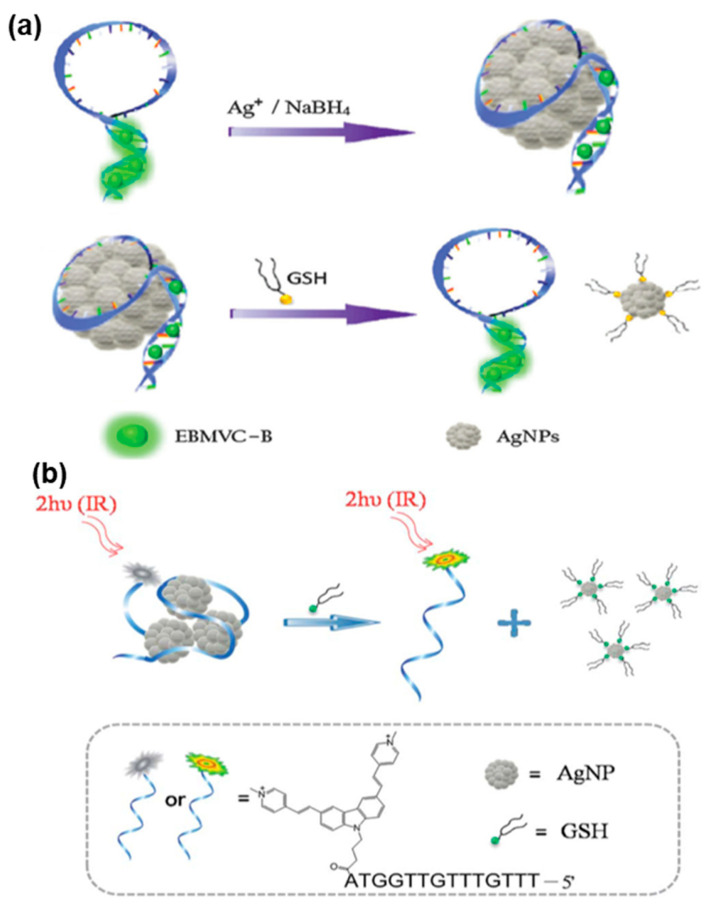
FNA probes based on TPE for biothiols analysis. (**a**) Schematic illustration of the AgNPs/DNA/TP dye nanoprobe for GSH detection. Adapted with permission from [91]. (**b**) Schematic illustration of the AgNPs/DNA-TP dye conjugate used for biothiols detection. Adapted with permission from [92].

As shown in Figure 4a, Tang et al. presented a TPE-based biosensor for GSH detection in living cells and tissues [91]. The sensor was prepared by forming DNA-templated silver nanoparticles (AgNPs) and binding TP dye to the DNA. The resulting conjugate, AgNPs/DNA/TP dye, exhibited favorable TP-sensitized fluorescence characteristics, excellent cell permeability and biocompatibility. Importantly, the conjugation of DNA/TP dye with DNA-templated AgNPs was achieved through a simple process that did not require surface functionalization or covalent labeling. By conducting assays in vitro and in vivo, the AgNPs/DNA/TP dye nanoprobe demonstrated the capability for quantitative GSH detection, even within complex biological environments.

Similarly, Liu et al. fabricated AgNPs/DNA-TP dye conjugates as a TPE-based nanoprobe for imaging biothiols in living cells (Figure 4b) [92]. The DNA-templated AgNPs served as biocompatible nanoplatforms for delivering DNA into living cells, while also effectively quenching fluorescence. When biothiols were present, such as GSH, the robust thiol–silver interaction led to the detachment of TP dye-labeled ssDNA from the surface of AgNPs. This detachment subsequently induced the fluorescence emission of the TP dye, allowing for the detection of biothiols.

### 2.4. Two-Photon Excited FNA Probes for ATP Analysis

Adenosine 5′-triphosphate (ATP), an essential cellular component present in all living organisms, serves as an indispensable intracellular energy source and plays a role as a signaling molecule associated with energy status [93,94,95]. It fulfills a wide range of functions in numerous biological processes, including the regulation of ion channels, modulation of signaling cascades, and facilitation of protein transport activities, as well as having involvement in DNA replication and transcription [96,97]. ATP homeostasis, which refers to the maintenance of constant cellular ATP concentrations, is critical for cell, tissue, and organ system functionality [98,99]. Deficiency in the ATP level is considered to be related to many diseases such as malignant tumors [100,101]; by determining the ATP content in cells, tissues, or bodily fluids, it becomes possible to assess the state of cellular energy metabolism and gain insights into cell vitality and health [102]. Consequently, monitoring the changes in ATP concentration both in vitro and in vivo holds significant research importance for understanding real-time biological processes [103,104].

**Figure 5:**
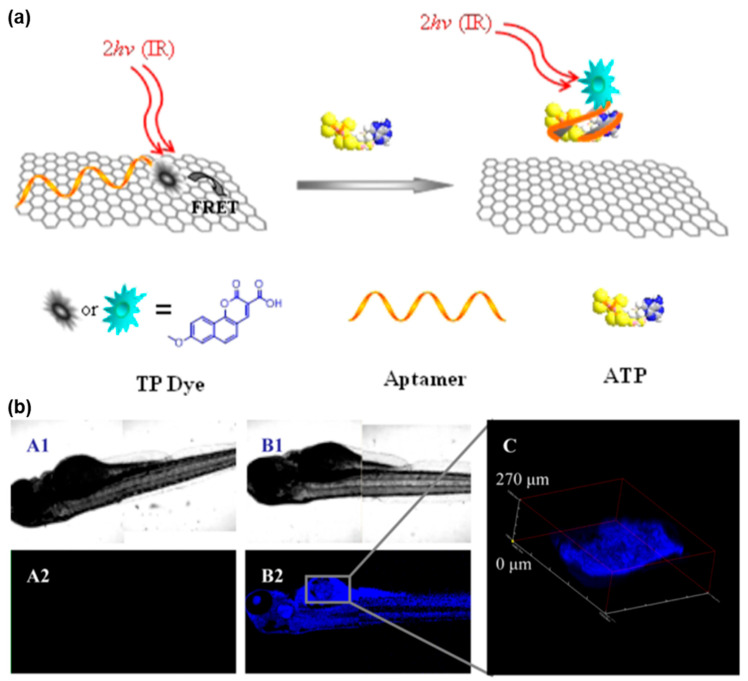
FNA probes based on TPE for ATP analysis. (**a**) Schematic illustration of TP GO/aptamer-based nanoprobe conjugate for ATP detection. (**b**) TP confocal microscopy of zebrafish treated with different probes. Adapted with permission from [105].

As shown in Figure 5, Yi et al. designed a TPE-based fluorescent nanoprobe, consisting of an ATP aptamer and TP dye, for molecular probing in various biological environments [105]. This approach harnessed the impressive quenching capability of graphene oxide (GO) towards nearby TP dyes. Notably, the binding affinity between ssDNA and GO surpassed that of the aptamer-target complex, facilitating the successful detection of ATP. The results revealed that the GO/aptamer-TP dye nanoprobe served as a sensitive, reliable, and selective sensor for quantitatively detecting ATP in complex biological environments. After efficient delivery into living cells or tissues, this nanoprobe acted as an in vivo sensor, enabling the specific and high-contrast TP imaging of ATP. This innovative design established a methodological foundation for the development of TPE-based fluorescent probes utilizing carbon nanomaterials to determine biologically relevant analytes in vitro or in vivo.

### 2.5. Two-Photon Excited FNA Probes for Metal Ions Analysis

Metal ions are vital components in biological systems, where they play critical roles in numerous biological processes [106,107,108]. Disruptions in metal homeostasis have been shown to be associated with many severe diseases, including various cancers, where irregular concentrations of metal ions are implicated [109]. Both excessive and deficient levels of metal ions can negatively impact their normal functions [110]. By quantifying the content of metal ions in samples, it becomes possible to assess the steady-state and dynamic changes of metal ions in cells [111], providing information about cellular metabolism, toxicity, and disease development [112]. Thus, there is significant interest in monitoring the concentrations of metal ions in living cells and their subcellular locations to gain insights into their roles in diverse human health and disease pathological processes [113,114,115].

**Figure 6:**
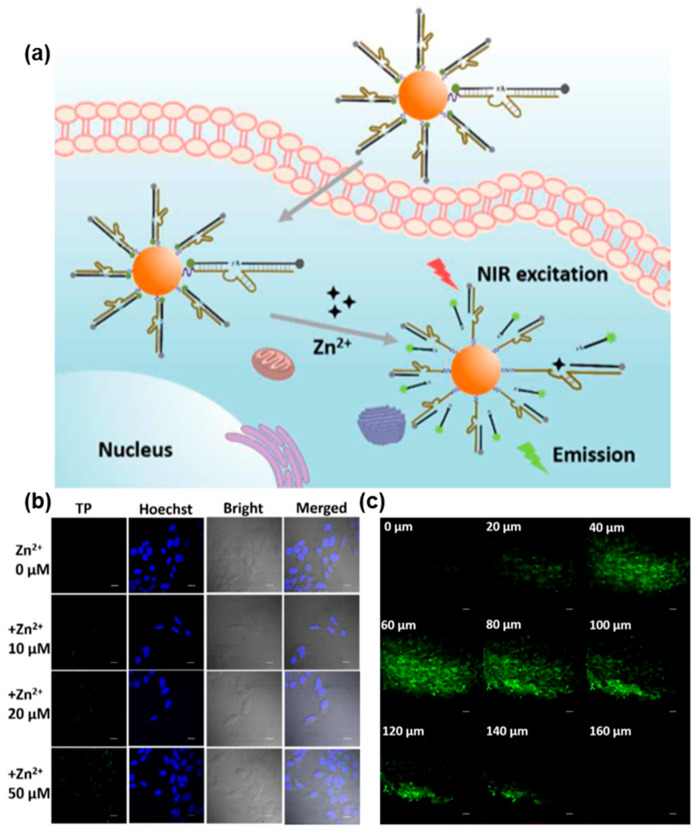
FNA probes based on TPE for metal ions analysis. (**a**) Schematic illustration of TPE-based fluorescent probe for selectively imaging intracellular Zn^2+^. (**b**) TP confocal microscopy images of intracellular different Zn^2+^ concentrations. (**c**) The TP fluorescence images of rat liver tissue with different penetration depths. Adapted with permission from [116].

As shown in Figure 6, Yang et al. developed an innovative FNA probe for the live cell imaging of Zn^2+^ by utilizing an RNA-cleaving DNAzyme coupled with TP fluorophores [116]. To facilitate cellular uptake, AuNPs were employed as effective transporters. The modified TP fluorophores exhibited outstanding TP excitation efficiency and photostability. In the absence of Zn^2+^, both AuNPs and a molecular quencher contributed to the quenching of TP fluorophores. However, the presence of Zn^2+^ facilitated the specific cleavage of the substrate strand labeled with TP fluorophores by the DNAzyme, resulting in enhanced fluorescence and TP imaging. Notably, the FNA probe demonstrated remarkable selectivity for Zn^2+^ in comparison to other metal ions commonly existing in biological environments. With the aid of TP fluorophores, the TP probe could be excited by NIR light, enabling deep tissue penetration of up to 160 µm for the imaging of Zn^2+^. This TP-based analysis strategy holds promise for the detection of various metal ions in biological systems, offering enhanced tissue penetration capabilities and reduced phototoxicity.

## 3. Two-Photon Activated FNA Probes for Biosensing

Light, as an external stimulus, can be instantly and accurately transmitted to specific locations. Additionally, it possesses different wavelengths and is thereby capable of inducing a wide range of chemical reactions [117,118]. The integration of a photocleavable (PC) linker into the DNA strand allows for the formation of light-activated structures, enabling the precise spatiotemporal activation of FNA probes through controlled light irradiation [119,120,121]. Therefore, FNA probes based on two-photon activation (TPA) exhibit exceptional properties, such as heightened temporal and spatial resolution, which enables them to be used as powerful tools for obtaining detailed information about analytes in living cells, especially in single cells [122]. Through single-cell detection using TPA-based FNA probes, we can understand the heterogeneity of different cells within the same sample and reveal the differences between cells in normal physiological or disease states. This method has important applications in biomedical research, clinical diagnostics, and personalized treatments.

### 3.1. Two-Photon-Activated FNA Probes for RNA Analysis

Traditional methods for RNA detection often necessitate a substantial number of cell samples, making it challenging to precisely measure RNA expression in heterogeneous cells [123,124]. However, the utilization of TPA-based FNA probes enables single-cell RNA detection, facilitating the quantitative analysis of RNA in individual cells. This approach unveils RNA differences between different cells, enhancing our comprehension of its roles in cellular functions, regulatory mechanisms, and disease progression [125,126,127]. The application of this technology extends to diverse fields including cancer research, neuroscience, immunology, and more, guiding personalized treatment and precision medicine and deepening our understanding of miRNA regulatory networks.

**Figure 7:**
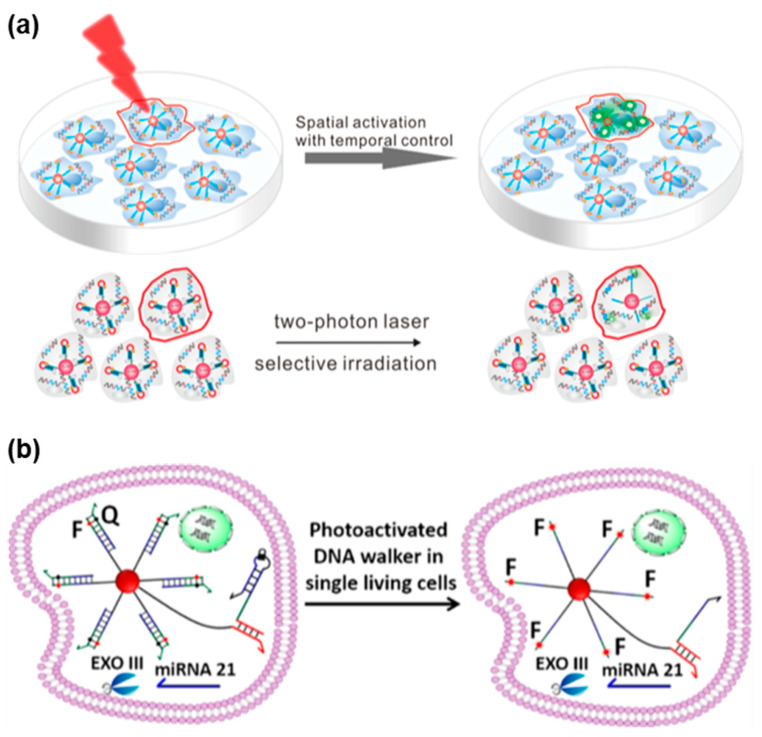
FNA probes based on TPA for RNA analysis. (**a**) Schematic illustration of TPA-based nanoflares for mRNA detection in the single living cells via selective TP irradiation at 740 nm. Adapted with permission from [128]. (**b**) Schematic illustration of TPA-based DNA walker probe for signal amplification miRNA imaging via selective TP irradiation at 740 nm. Adapted with permission from [129].

As shown in Figure 7a, Lin et al. designed TPA-based nanoflares that enabled high spatiotemporal control for monitoring intracellular mRNA [128]. Such nanoflares comprised light-responsive DNA hairpin probes and AuNPs. In the absence of UV irradiation, the DNA hairpin could remain awakened, unresponsive to the target analyte. Upon UV activation, the hairpin structures were disrupted, revealing sticky domains that acted as toeholds for initiating strand displacement reactions, eventually leading to the release of fluorophores and subsequent fluorescence enhancement. With the ability to adjust light irradiation, temporal control over mRNA detection in living cells was achieved using TPA-based nanoflares. Under TP laser irradiation, these nanoflares could selectively detect mRNA in the single living cell at desired time points. In contrast to the conventional nanoflares, the novel TPA-based nanoflares exhibited heightened detection sensitivity and enabled the detection of biomarkers in a single cell with precise spatiotemporal control.

Chen et al. devised a TPA-based DNA walker probe that enabled light-controllable signal amplification for imaging cancer-related miRNA in a single living cell (Figure 7b) [129]. This developed probe combined a light-activated nucleic acid strand displacement reaction with the traditional exonuclease III (EXO III)-assisted DNA walker, utilizing DNA nanoflares as the foundation. The researchers successfully demonstrated the amplification imaging of light-activated signals for miRNA-21 in individual living HeLa cells via selective TP irradiation (λ = 740 nm) using femtosecond laser-equipped confocal microscopy.

### 3.2. Two-Photon-Activated FNA Probes for ATP Analysis

As with the previously mentioned RNA assays, traditional ATP detection methods also face the issue of requiring a large amount of sample, which masks the heterogeneity between cells [130]. However, using TPA-based FNA probes for single-cell ATP detection allows for the precise measurement of ATP content in individual cells, revealing variations in ATP levels among different cells. This helps us understand the functional features of cells, metabolic states, and differences between cell types [131]. Through single-cell detection, we can gain insight into cellular energy metabolism, including ATP synthesis pathways, energy production, and consumption balance, among others. This contributes to our understanding of cellular energy regulation mechanisms and the changes in energy demand during different physiological or diseased states [132]. In a word, single-cell ATP detection holds great significance in elucidating cellular energy metabolism, uncovering cell heterogeneity, investigating disease mechanisms, and advancing precision medicine.

**Figure 8:**
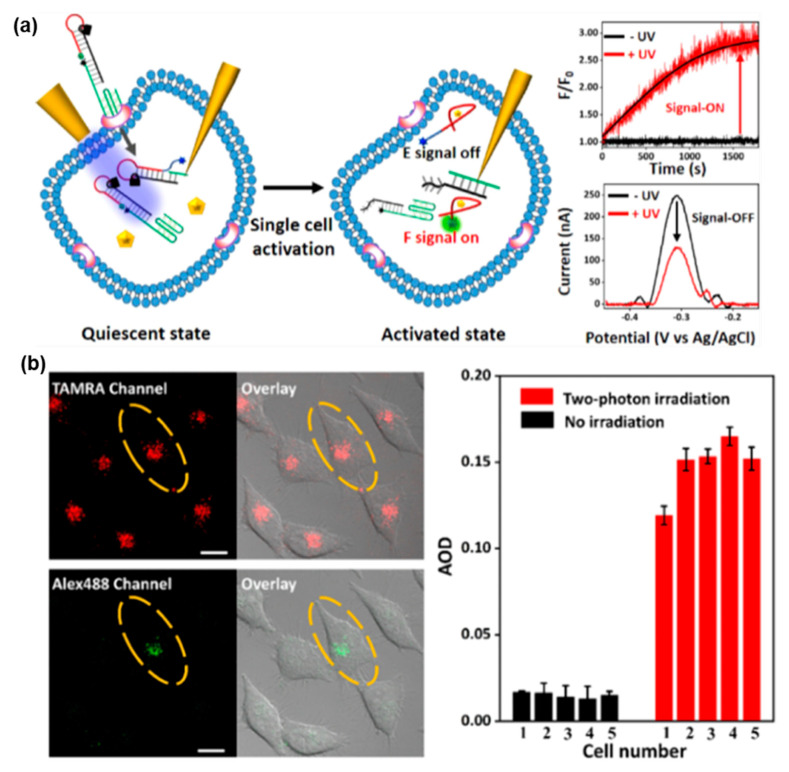
FNA probe based on TPA for ATP analysis. (**a**) Schematic illustration of light-activated FNA probe for the fluorescence and electrochemical bimodal imaging of ATP in a single living cell. (**b**) The fluorescence signal of a single living cell vis TP irradiation at 740 nm. Adapted with permission from [133].

As shown in Figure 8, Duan et al. introduced a light-activated FNA probe constructed with a PC linker for ATP detection in a single living cell [133]. Two distinct methods for spatiotemporal activation of the probe were discussed. The first method involved the use of a micrometer-sized optical fiber to selectively direct UV light (λ = 365 nm) for activating the light-activated FNA probe in living cells. Alternatively, a TP laser confocal scanning microscope was employed as the second method to selectively activate the probe via TP irradiation (λ = 740 nm) in single living cells. The aptamer sequence integrated into the light-activated FNA probe selectively interacted with ATP, leading to the generation of the indicated signal. With TP irradiation, the developed light-activated biosensor enabled high spatiotemporal resolution for ATP detection in single living cells at a desired time and location. These findings highlight the potential of TPA-based FNA probes for monitoring biomarkers at different cell stages.

## 4. Conclusions and Future Prospects

Typically, TP-based FNA probes are composed of FNA sequences along with fluorophores or linkers that are sensitive to TP irradiation. The FNA sequences serve a crucial role in specific interactions with target biomolecules and can include aptamers, DNAzymes, modified nucleic acids, and other related elements. TP technology serves a dual purpose in TP-based FNA probes. Not only does it function as an excitation light source for TP dye, producing fluorescence signals, but it can also serve as an activation light source, enabling spatiotemporal control over FNA probes. As a result, TP-based FNA probes offer advantages such as high spatiotemporal resolution, deep tissue penetration, low photodamage, and high sensitivity. These probes have garnered significant attention in the field of studying molecular interactions in cells, monitoring and imaging drug delivery processes, and quantifying biomarkers. In addition, they are also expected to be used in the regulation of biomimetic materials, the synthesis of artificial tissues, and the preparation of biological hydrogels.

Despite the years of development and utilization of TP-based FNA probes in biomedicine, they are still considered to be in their early stages. For the further development of high-performance TP-based FNA probes and the promotion of their commercialization, there are still some limitations with TP-based FNA probes that should be addressed. Priority should be given to the development of TP dye molecules with higher TP excitation efficiency and environmental stability, thereby improving the imaging resolution and reliability. Additionally, there is a need to develop a user-friendly TP platform that can achieve different regulation effects by adjusting the wavelength, power, and spot size of the two-photon laser. Lastly, efforts should focus on developing additional FNA probe vectors to improve their delivery and targeting efficiency.

## Data Availability

Not applicable.
